# Regulation of *Plasmodium falciparum* Glideosome Associated Protein 45 (PfGAP45) Phosphorylation

**DOI:** 10.1371/journal.pone.0035855

**Published:** 2012-04-27

**Authors:** Divya Catherine Thomas, Anwar Ahmed, Tim Wolf Gilberger, Pushkar Sharma

**Affiliations:** 1 Eukaryotic Gene Expression Laboratory, National Institute of Immunology, New Delhi, India; 2 Department of Molecular Parasitology, Bernhard-Nocht-Institute for Tropical Medicine, Hamburg, Germany; 3 M. G. DeGroote Institute for Infectious Disease Research and Department of Pathology and Molecular Medicine, McMaster University, Hamilton, Canada; University of Oklahoma Health Sciences Center, United States of America

## Abstract

The actomyosin motor complex of the glideosome provides the force needed by apicomplexan parasites such as *Toxoplasma gondii (Tg)* and *Plasmodium falciparum (Pf)* to invade their host cells and for gliding motility of their motile forms. Glideosome Associated Protein 45 (PfGAP45) is an essential component of the glideosome complex as it facilitates anchoring and effective functioning of the motor. Dissection of events that regulate PfGAP45 may provide insights into how the motor and the glideosome operate. We found that PfGAP45 is phosphorylated in response to Phospholipase C (PLC) and calcium signaling. It is phosphorylated by *P. falciparum* kinases Protein Kinase B (PfPKB) and Calcium Dependent Protein Kinase 1 (PfCDPK1), which are calcium dependent enzymes, at S89, S103 and S149. The Phospholipase C pathway influenced the phosphorylation of S103 and S149. The phosphorylation of PfGAP45 at these sites is differentially regulated during parasite development. The localization of PfGAP45 and its association may be independent of the phosphorylation of these sites. PfGAP45 regulation in response to calcium fits in well with the previously described role of calcium in host cell invasion by malaria parasite.

## Introduction

Malaria is one of the major causes of morbidity and mortality in the developing world claiming as many as 1 million lives per year [1]. *Plasmodium falciparum* causes the most severe form of the disease. It follows a complex life cycle involving different stages of development that require human and mosquito hosts.

Invasion of an erythrocyte by a merozoite is initiated by specific ligand receptor interactions between them [2]. Following this, the merozoite reorients and juxtaposes its apical end with the Red Blood Corpuscle (RBC) membrane resulting in the formation of an irreversible tight junction. The parasite then actively propels itself into the RBC membrane and subsequently is enclosed inside the parasitophorous vacuole. The motor complex which is engaged in the invasion of merozoites is known as the glideosome and was initially described in *Toxoplasma gondii* [3], [4]. It comprises of a heterotetrameric complex that is anchored in the Inner Membrane Complex (IMC) of the zoite pellicle. In *P. falciparum*, it has been demonstrated to comprise of a class XIV Myosin (PfMyoA) and its associated light chain Myosin Tail Interacting Protein (PfMTIP) [5]–[8]. PfGAP45 is a 204 amino acid protein, which is highly conserved. It is predicted to consist of two separate domains, an N terminal coiled coil domain and a C terminal globular domain. Glideosome Associated Protein 50 (PfGAP50) is a transmembrane protein that anchors the glideosome in the IMC [8], [9]. Gliding motility is achieved by the controlled polymerization of actin filaments between the Plasma Membrane (PM) and the IMC [10]. The actin-myosin motor is in turn connected to extracellular ligands that interact with receptors on the erythrocyte membrane. The forward motion of the parasite is caused by repetitive binding and release of actin filaments by myosin. Post translational modifications have been reported to occur on different players of the glideosome complex. PfMTIP was shown to be phosphorylated in the parasite and is an *in vitro* substrate for PfCDPK1 [7], [11]. TgGAP50 requires glycosylation at three different sites in order to be targeted to the IMC [12]. TgGAP45 has been implicated in the recruitment of the motor complex as well as in the maintenance of pellicle cohesion. The N terminus of TgGAP45 has putative acyl modification sites and mutational analysis indicated that these may be essential for temporally regulating the insertion of the protein into the IMC. In addition, the palmitoylation of cysteines at the C-terminus of TgGAP45 was implicated in anchoring it to the outer leaflet of the IMC [13]. PfGAP45 is also myristoylated and palmitoylated and these modifications may be essential for its membrane targeting [14]. GAP45 undergoes phosphorylation in both *Toxoplasma* and *Plasmodium* [7], [15]–[17].

Given the importance of PfGAP45 in motor function and invasion, it is important to understand cellular processes that may regulate its phosphorylation. Calcium release mediated by PLC [17]–[19] and Cyclic Adenosine Phosphate Ribose-Ryanodine Receptor (cADPR-RyR) pathways [20] has been implicated in erythrocyte invasion by the parasite. Our studies demonstrate that the phosphorylation of PfGAP45 is regulated by calcium and is controlled by PLC. We have identified that PfGAP45 is phosphorylated at S103 and S149 in response to PLC mediated calcium release and phosphorylation of PfGAP45 at these sites is developmentally regulated. While PfPKB can phosphorylate PfGAP45 on S103, PfCDPK1 can also phosphorylate it on S89 and S149. Both PfPKB and PfCDPK1 are regulated by calcium; while calcium directly stimulates PfCDPK1 activity [7], it can combine with calmodulin to activate PfPKB [21].

**Figure 1:**
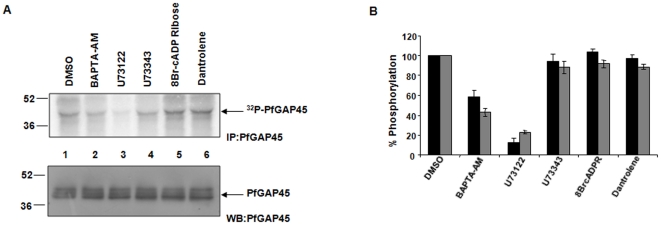
PfGAP45 phosphorylation is regulated via the PLC pathway in the parasite. A. [^32^P] orthophosphoric acid was used to metabolically label synchronized parasites treated with 10 µM U73122 or its inactive analogue U73343, 40 µM 8-Br-cADP-ribose or 10 µM Dantrolene. Immunoprecipitation was carried out using anti-GAP45 antibody and the IPs were electrophoresed and analyzed by phosphorimaging. *Lower panel*, western blot for PfGAP45 was performed on the protein lysate used in the above immunoprecipitation experiments. B. The phosphate incorporation in PfGAP45 in experiments like the one described in panel A was assessed by either densitometric quantitation or by cerenkov counting of bands corresponding to ^32^P-labelled PfGAP45, which was normalized to total PfGAP45 levels. % phosphorylation of PfGAP45 in response to various treatments is provided, the phoshorylation of DMSO treated control was taken as 100%. The mean from the densitometry measurements (black bars) for four independent experiments is shown. For two of these experiments, cerenkov counting of labeled PfGAP45 band was also performed and the average is presented as grey bars, error bars represent standard error.

## Materials and Methods

### Parasite culture and transfections

For all experiments, the 3D7 strain of *P. falciparum* was used, which was cultured in complete RPMI 1640 medium with 5% albumax (Invitrogen) at 37°C as described previously [22]. Parasites were synchronized by sorbitol treatment [22]. For generating transgenic lines expressing PfGAP45 fused with Green Fluorescent Protein (GFP) at its C-terminus, PfGAP45 and its mutants were cloned in pARL vector [23] containing the GFP gene and a human Dihydrofolate Reductase (DHFR) gene which confers resistance to WR99210. The following primers were used Forward: 5′GGGGTACCGGATGGGAAATAAATGTTCA3′, Reverse: 5′ CCG CCTAGGATTAGCTCAATAAAGGTG 3′ to facilitate cloning in KpnI/AvrII sites of the pARL-GFP vector. The transfection of plasmid DNA in the parasite was done by electroporation [24] and selected with WR99210 [25].

**Figure 2:**
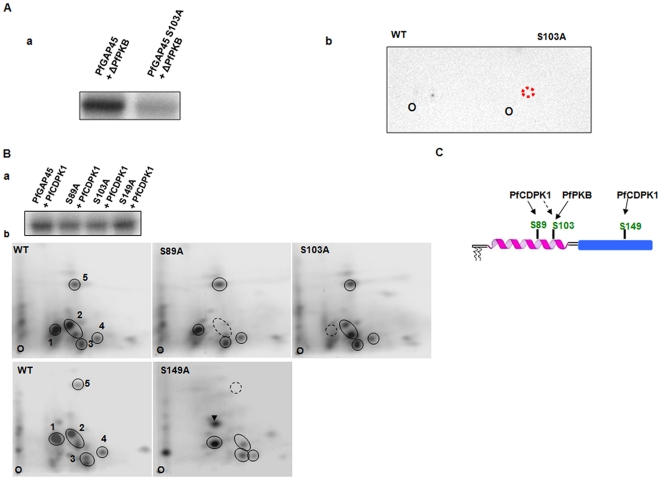
PfGAP45 is phosphorylated by PfPKB and PfCDPK1. **A**, a *In vitro* kinase assay was performed using recombinant ΔPfPKB to phosphorylate an equal amount of PfGAP45 or its S103A mutant. The phosphorimage shows phosphate incorporation in PfGAP45. *b*, Phosphopeptide mapping was performed on ΔPfPKB phosphorylated PfGAP45 or the S103A mutant. **B.** *a*, PfCDPK1 kinase assay was performed using an equal amount of recombinant PfGAP45 or its S89A, S103A and S149A mutants. *b*, Phosphopeptide mapping was carried out on PfGAP45 or indicated mutants which were phosphorylated with PfCDPK1. Five major spots representing labeled phosphopeptides in the case of WT PfGAP45 are encircled and are numbered 1–5. Broken circles are used to indicate the lack or reduced labeling of peptides in the case of mutants. Arrowhead indicates the additional phosphopeptide in S149A mutant. The origin where the peptides were spotted is marked as “O”. **C.** PfGAP45 is phosphorylated at least three major sites. While S89 and S149 can be phosphorylated by PfCDPK1 *in vitro*, PfPKB as well as PfCDPK1 phosphorylate S103. S89 and S103 are present in the coiled-coil region of the kinase whereas S149 is in the globular domain of PfGAP45.

### Recombinant protein expression and generation of antisera

GST-ΔPfPKB [26], 6xHis-GAP45 and 6xHis-MTIP [17] were expressed and purified as previously described. Mutations in PfGAP45 were made using either Quickchange™ Site-Directed Mutagenesis Kit (Stratagene) or by overlapping PCR. PfCDPK1 was cloned using forward primer 5′CGTGGATCCATGGGGTGTTCACAAAGTTCAAACG 3′ and reverse primer 5′CCGCTCGAGTTGAAGATTTATTATCACAAA 3′ in pET28a vector. The 6x-His tagged protein was expressed in BL21 RIL (DE3) *E.coli* cells by using 1 mM Isopropyl-1-thio-β-D-Galactopyranoside (IPTG) at 18°C for 16 hours. Cells were resuspended in cold resuspension buffer (50 mM PO_4_ buffer, 150 mM NaCl, 0.1% Nonidet-P40, 1 mM DTT and 10 µg/µl pepstatin, 10 µg/µl leupeptin, 1 mM benzamidine, 1 mM PMSF at pH 7.4), sonicated on ice and centrifuged at 12000 g for 30 minutes at 4°C. The cell lysate was incubated with equilibrated Ni-NTA agarose beads (Invitrogen) at 4°C for 4 hours. Following binding, the resin was washed with resuspension buffer and the protein was eluted using 50–300 mM of imidazole. The eluted fractions were dialyzed against 50 mM sodium phosphate buffer pH 7.5, 10% glycerol and 1 mM DTT.

**Figure 3:**
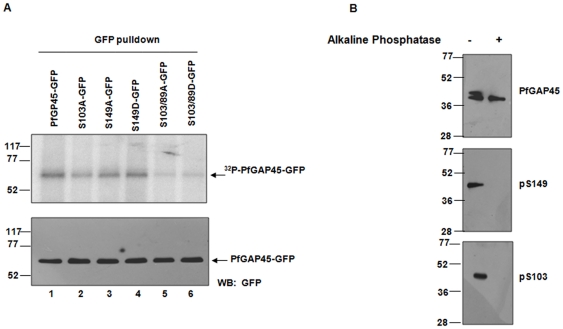
PfGAP45 is phosphorylated at S89, S103 and S149 in the parasite. **A.** Transgenic parasite lines expressing GAP45 or various mutants fused to GFP were generated (see Materials and Methods). Schizont stage parasite cultures were incubated with [^32^P] orthophosphoric acid. Using anti-GFP antibody, GAP45 or its mutants were immunoprecipitated followed by SDS PAGE and phosphorimaging. *Lower Panel*, western blotting was performed on the protein lysates from the above experiment using anti-GFP antibody to assess the levels of PfGAP45-GFP. **B.** Antisera were raised against phosphopeptides corresponding to S103 (bottom panel) and S149 (middle panel) (see Experimental procedures, Fig. S1). Using these and antisera against total PfGAP45 (top panel), western blotting was performed on schizont lysate, which was either left untreated or treated with alkaline phosphatase. Both phospho-antibodies recognized a band corresponding to PfGAP45 only in the untreated parasites indicating that S103 and S149 are phosphorylated in the parasite.

### Generation of antisera and phosphorylation-site specific antibodies

Antisera were raised against recombinant PfGAP45 and PfMTIP, which has been described previously [17]. The antibodies against PfGAP45 phosphorylated at S103 or S149 were custom generated by Antagene. Inc. (USA). For this purpose, peptides with the following sequence were used: pS103: DLERSN-pS-DIYSES; pS149: EPAHEE-pS-IYFTY. The phosphorylated peptides were used to immunize rabbits over a 10 week schedule. Antisera were collected and antibody titers were determined by performing ELISA against the phosphorylated and the unmodified peptides. The antisera were enriched in the anti phosphopeptide antibodies by using a phosphopeptide affinity column and were further purified by passing through an unmodified peptide column by affinity depletion.

**Figure 4:**
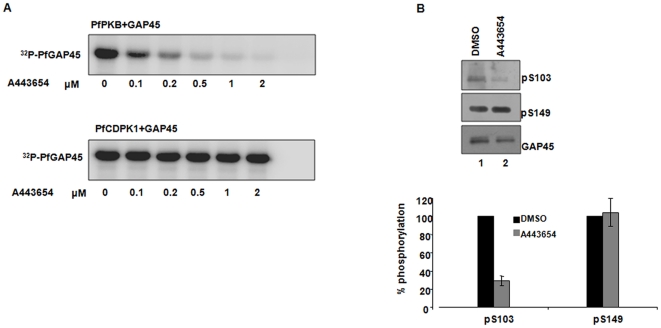
PfGAP45 may be phosphorylated by PfPKB at S103 in the parasite. **A.** A443654 inhibits PfPKB and not PfCDPK1. *In vitro* kinase assays were performed to phosphorylate recombinant PfGAP45 by ΔPfPKB or PfCDPK1 in the absence (DMSO) or presence of indicated concentration of A443654. Treatment with A443654 inhibited the activity of PfPKB while PfCDPK1 activity remained almost unaffected. **B.** A443654 blocks the phosphorylation of S103 in the parasite. Western blotting was performed using anti-phospho-S103 and anti-phospho-S149 antisera on the lysate of schizonts that were treated with A443654 (lane 2) or DMSO (lane 1). While almost no change in the phosphorylation levels of S149 was seen, phosphorylation at S103 was significantly reduced. *Lower panel*, The densitometric quantitation of pS103-GAP45 and pS149-GAP45 bands was performed and normalized with respect to GAP45. The % phosphorylation of S103 or S149 in comparison to DMSO (100%) is shown and the mean of two independent experiments is provided and error bars represent standard error.

### Kinase assays

Using recombinant PfGAP45 or its mutants and ΔPfPKB or PfCDPK1 as kinase, activity assays were carried out in a reaction buffer containing 50 mM Tris pH 7.5, 10 mM magnesium chloride, 1 mM dithiothreitol and 100 µM Adenosine triphosphate (ATP) (15 µCi/reaction) For reactions that were carried out using PfCDPK1 as enzyme 0.1 mM CaCl_2_ was added additionally to the reaction mix. Reactions were carried out at 30°C for 40 minutes and stopped by boiling in SDS-PAGE loading buffer. The reaction mixture was electrophoresed on SDS-PAGE and phosphorylation of proteins was detected by phosphorimaging using a Fuji FLA5000 scanner. For inhibition assays, A443654 was incubated with the enzyme for 10 minutes prior to the addition of the substrate and radiolabelled ATP.

**Figure 5:**
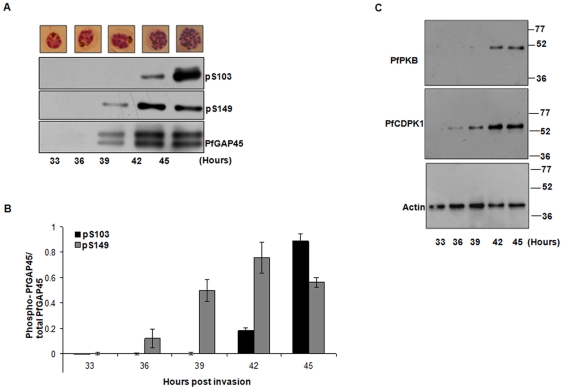
Phosphorylation of S103 and S149 on PfGAP45 is temporally regulated. **A.** Parasites were tightly synchronized and were harvested at indicated time post-invasion (late trophozoite to segmentor) to prepare protein lysates. Western blotting was carried out using anti-PfGAP45, anti-phospho S103 and anti-phospho S149 on these lysates. **B.** The intensity of the bands in the western blots in panel A was quantified by densitometry, which was performed using Image J software. The ratio of phospho-S103 or phospho-S149 to the total PfGAP45 is indicated in the graph. Average from two different experiments is provided, the error bars represent standard error. **C.** Western blotting was performed on protein lysates from tightly synchronized parasites from the experiment described in panel A using anti-PfPKB and anti-PfCDPK1 antibodies. Actin was used as the loading control.

### Phosphopeptide mapping

The kinase assays were carried out as described above and the reaction mixture was transferred to nitrocellulose membrane following SDS-PAGE. The radiolabelled bands corresponding to phosphorylated PfGAP45 or its mutants were excised from the membrane and washed before blocking with 0.5% polyvinylpyrrolidine (prepared in 100 mM acetic acid) for 30 minutes at 37°C with shaking. After washing with 50 mM ammonium bicarbonate pH 8.0, overnight digestions were carried out with trypsin gold (Promega). The supernatants from each reaction were collected and dried using a speedvac at 45°C. The phosphopeptides were resupended in 10 µl 2.5% formic acid, 7.8% glacial acetic acid pH 1.9 and counts per minute (cpm) in each sample were determined by scintillation counting. Equal cpm from each sample were loaded onto PEI cellulose TLC plates (Merck). Phosphopeptides were separated in the first dimension by electrophoresis at pH 1.9 for 50 minutes at 1000 V using Hunter thin layer peptide mapping system (CBS Scientific. Inc.). Separation in the second dimension was carried out by chromatography in 7.5% glacial acetic acid, 25% pyridine, 37.5% n-butanol. The radiolabelled peptides were detected by phosphorimaging of the TLC plates [27].

**Figure 6:**
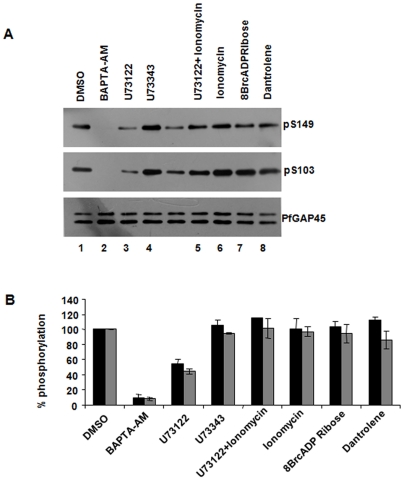
Phosphorylation at both S103 and S149 is regulated via the PLC pathway. **A.** Schizonts were treated with 40 µM BAPTA-AM, 10 µM U73122 or its analogue U73343, 5 µM Ionomycin alone or in combination with 10 µM U73122, 40 µM 8Br cADP-ribose and 10 µM Dantrolene followed by Western blotting using anti-phospho S103 and anti-phospho S149 antisera. The phosphorylation at both S103 and S149 seems to be regulated via the PLC pathway. **B.** Densitometric quantitation of indicated bands from the experiments described in panel A. The % phosphorylation of S103 (black) or S149 (grey) in comparison to DMSO (100%) is provided. The mean of two independent experiments is shown and error bars represent standard error

### Immunoprecipitation

Parasite-infected erythrocytes after various treatments were lysed in 0.05% saponin at 4°C for 10 minutes. The released parasites were washed in cold Phosphate Buffered Saline (PBS) and resuspended in complete lysis buffer containing 100 mM Tris pH 7.5, 100 mM sodium chloride, 5 mM EDTA, 1% Triton X-100, 100 µM sodium orthovanadate, 20 µM β glycerophosphate, 1× protease inhibitor cocktail (Roche) and 10% glycerol. 100 µg protein lysate was used for immunoprecipitation and incubated with relevant antibodies at 4°C for 5 hours followed by incubation with protein A+G sepharose beads (GE Healthcare) for 12 hours. The beads were washed with cold lysis buffer and resuspended in 50 µl of kinase assay buffer or SDS PAGE loading buffer.

**Figure 7:**
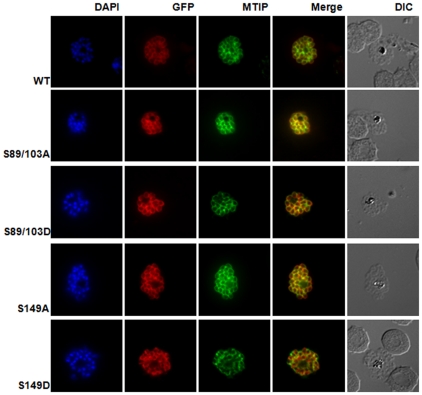
PfGAP45 phosphorylation mutants co-localize with the IMC marker PfMTIP. Immunofluorescence assays were performed using anti-GFP (red) and anti-PfMTIP (green) antibodies on parasite lines over-expressing PfGAP45-GFP or its variants. Both WT and mutant PfGAP45 show co-localization with PfMTIP around the periphery of merozoites in mature schizonts. Similar results were obtained upon live imaging of GFP fluorescence in parasites (Fig. S3).

### Immunofluorescence assays (IFA)

IFA was performed on either thin blood smears or on parasites in suspension. For IFAs performed on smears, cold methanol was used for fixation. The parasites were permeabilized with 0.05% saponin in PBS followed by washing in PBS and blocked with 3% bovine serum albumin for 30 minutes at room temperature. The parasites were incubated with relevant antibodies at 4°C, washed with PBS and incubated with AlexaFluor 488/594 labelled secondary antibodies (Invitrogen) at room temperature. Following washing, vectashield mounting medium containing DAPI was used. In the case of IFAs on parasite suspensions, 4% paraformaldehyde and 0.0075% glutaraldehyde in PBS pH 7.4 was used for fixation and the parasites were permeabilized with 0.1% Triton ×100 [28]. The stained parasites were visualized using AxioImager Z1 microscope (Carl Zeiss). Z stacking during image acquisition and processing of images was done using Axiovision 4.8.2 software. The Z-stacks which best represented the immunolocalization were used for illustrations in the figures. Adobe photoshop software was also used for preparing images for figures.

**Figure 8:**
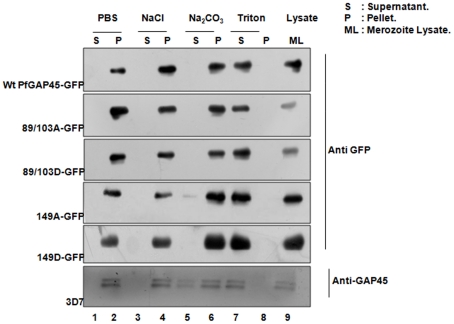
The role of phosphorylation on membrane association of PfGAP45. The solubility of PfGAP45-GFP or its mutants and endogenous PfGAP45 was assessed by performing extractions in PBS, PBS/1 M NaCl, PBS/0.1 M Na_2_CO_3_ pH 11.0 or PBS/0.1% Triton ×100. The supernatant (S) and the pellet (P) fractions were subjected to western blotting using anti GFP and anti GAP45 antibody (bottom panel) as indicated.

### Fractionation of merozoite proteins

Segmentor/schizont stage, which contained more than ∼18 merozites, was centrifuged at ∼300 g for 5 minutes to separate erythrocytes from free merozoites. The supernatant containing merozoites was further centrifuged at 15000 g for 10 minutes to collect free merozoites. To the merozoite pellet, either PBS, PBS/1 M sodium chloride, PBS/0.1 M sodium carbonate pH 11.0 or PBS/0.1% triton X-100 was added and lysed by passing the suspension through a syringe and freeze thawing in liquid nitrogen followed by centrifugation at 16000 g for 1 hour at 4°C. The pellet fraction in all cases was solubilized in 2% SDS [13].

### Alkaline phosphatase treatment of lysate

30 µg of segmentor schizont lysate from tightly synchronized culture was treated with 60 units (2 units/µg protein) alkaline phosphatase in a 50 µl reaction at 37°C for 2 hours. Reactions were stopped by boiling in SDS PAGE loading buffer and used for western blotting.

### Metabolic labeling

Tightly synchronized cultures at ∼42 h post invasion at ∼8% parasitemia/5% hematocrit were washed with phosphate or methionine/cysteine deficient RPMI 1640 medium (Hyclone) supplemented with 5% albumax. Parasites in culture were incubated with [^32^P] orthophosphoric acid (1–2 mCi/ml) [29] or [^35^S] methionine/cysteine (300 µCi/µl) [5], [14] till most parasites were segmentors. Subsequently, parasite lysates were made and relevant antibodies were used for immunoprecipitation as described above. The immunoprecipitates (IP) were electrophoresed and labelled proteins were detected using a Fuji FLA5000 scanner or autoradiography. The phosphate incorporation was quantitated by performing densitometry using the Image J software.

## Results

### PfGAP45 is phosphorylated in response to phospholipase C mediated calcium release

We [17] and others [7] have previously reported that PfGAP45 is phosphorylated in the parasite. However, signaling pathways involved in its phosphorylation have remained obscure. Since two calcium dependent kinases, PfPKB [17], [21] and PfCDPK1 [7], [30], phosphorylate PfGAP45 *in vitr*o, we first probed if calcium plays a role in PfGAP45 phosphorylation in the parasite. Parasite proteins were labeled with ^32^P followed by immunuprecipitation of PfGAP45 (Fig. 1, Fig. S4). PfGAP45 was phosphorylated in the parasite and treatment of parasites with BAPTA-AM, an intracellular calcium chelator, significantly reduced PfGAP45 phosphorylation (Fig. 1, lane 2), which suggested that intracellular calcium regulates PfGAP45 phosphorylation. In most eukaryotic cells, calcium release from intracellular stores is triggered by two major pathways; PLC triggered calcium release involves Inositol triphosphate (IP3) receptors and cADP-ribose stimulates calcium release via ryanodine-responsive calcium channels [31]. Although the IP3 receptor and RyR channels have not been identified in the parasite, both of these pathways regulate calcium release in the parasite and in the invasion of host RBCs by the malaria parasite [17], [20]. To assess the role of PLC in PfGAP45 phosphorylation, parasites were treated with U73122, a PLC inhibitor or its inactive analogue U73343. While U73122 caused a marked reduction in PfGAP45 phosphorylation, U73343 was significantly ineffective indicating that PLC mediated calcium release may promote PfGAP45 phosphorylation (Fig. 1, lane 3 v/s lane 4). In contrast, 8Br-cADP-ribose or Dantrolene, which were used to explore the involvement of cADPR-RyR pathway, failed to cause any significant change in PfGAP45 phosphorylation (Fig. 1, lanes 5 and 6).

### Identification of PfPKB and PfCDPK1 phosphorylation sites on PfGAP45

The finding that PfGAP45 is a target of calcium signaling in the parasite fits in well with previous studies which indicated that PfGAP45 can be phosphorylated by two calcium regulated kinases PfPKB [17] and PfCDPK1 [7] *in vitro*. Since the identity of the target phosphorylation sites for these kinases remained unknown, efforts were made to address this important question. Recombinant PfGAP45 was incubated with a constitutively active PfPKB mutant, ΔPfPKB or PfCDPK1 and subjected to mass spectrometry as part of an independent study (Sharma, Jaffe, unpublished data). Mass spectrometric studies lead to the identification of phosphopeptides from the kinase assay reactions and suggested a single putative phosphorylation site (S103) for PfPKB and at least three sites (S89, S103 and S149) for PfCDPK1. To confirm the identity of these sites, S to A mutants were generated for these residues and the mutants were analyzed by phosphopeptide mapping. PfPKB phosphorylated PfGAP45 at one major site as indicated by a single labeled spot on the phosphopeptide map (Fig. 2A). The labeling of this phosphopeptide was abolished upon mutation of S103 to alanine (Fig. 2A, b) confirming it to be the phosphorylation site for PfPKB. In the case of PfCDPK1, at least five major phosphopeptides were generated on PfGAP45 (Fig. 2B, b). The kinase assay with mutants S89A, S103A and S149A revealed a decrease in the phosphorylation of S89 and S103 (Fig. 2B, a). The peptide maps revealed that the labeling of peptide 2 and 1 was abolished upon mutation of S89 and S103 respectively (Fig. 2B, b), which confirmed these two serines as phosphorylation sites for PfCDPK1. The mutation of S149A did not reveal a net decrease in phosphorylation on SDS-PAGE gel (Fig. 2B, a). Interestingly, the mutation of S149A resulted in slight rearrangement of the map: while a loss in the labeling for peptide 5 and a decrease for peptide 2 was evident, an additional labeled peptide emerged (Fig. 2B, b, arrowhead). This observation might explain no discernable decrease on net phosphorylation of S149A mutant (Fig. 2B, a). These data also suggested that S149 may be a phosphorylation site for PfCDPK1 and its phosphorylation possibly influences the phosphorylation of sites, which need to be identified. Furthermore, independent experiments using antibodies specific to PfGAP45 phosphorylated at S103 or S149 (described below) confirmed that S103 and S149 can be phosphorylated by PfCDPK1 *in vitro* (Fig. S1). Collectively, these results suggest that S103 can be phosphorylated by both PfPKB and PfCDPK1 and S89 and S149 are phosphorylated by PfCDPK1 (Fig. 2C).

### S89, S103 and S149 are phosphorylated in the parasite

Having demonstrated that PfGAP45 is phosphorylated at S89, S103 and S149 by PfCDPK1 and PfPKB *in vitro*, we investigated if these sites were phosphorylated in the parasite. To this end, parasite lines expressing wild type or mutant GAP45 with GFP fused to its C-terminus were generated. After metabolic labeling of parasite cultures with ^32^P, GFP fusion proteins were immunoprecipitated. A significant reduction in the phosphorylation of S103A was observed (Fig. 3A, lane 2), which was even more pronounced in the case of S89/S103A and S89/S103D double mutants (Fig. 3A, lanes 5 and 6). In comparison, S149A mutation caused only a minor decrease, which may have been due to a compensatory increase in total phosphorylation by phosphorylation at additional sites (Fig. 3A, lane 3) as indicated by *in vitro* experiments (Fig. 2B).

Phosphorylation site specific antibodies are useful tools to study site-specific phosphorylation of proteins *in vivo*. Antibodies against phosphopeptides corresponding to S103 and S149 were successfully generated (Figure S1). The western blot performed on parasite lysates with these antibodies resulted in a band corresponding to the size of PfGAP45 (Fig. 3B), Treatment of the parasite lysate with alkaline phosphatase resulted in a loss of immunoreactivity against these peptides suggesting that these antibodies specifically recognize PfGAP45 phosphorylated at S103 and S149 and confirmed PfGAP45 phosphorylation at these residues in the parasite.

### PfPKB, and not PfCDPK1, may phosphorylate PfGAP45 at S103 in the parasite

The results of kinase assays described above suggested that both PfCDPK1 and PfPKB may phosphorylate PfGAP45 at S103. We made attempts to identify the likely kinase that may phosphorylate S103 in the parasite. A443654, which was first identified as an inhibitor of mammalian PKB [32], inhibits PfPKB [17]. Unlike PfPKB, this inhibitor failed to inhibit PfCDPK1 activity *in vitro* suggesting that it is specific to PfPKB (Fig. 4A). When this inhibitor was added to parasites, a significant decrease in the phosphorylation of S103 was observed implicating PfPKB in the phosphorylation of PfGAP45 at S103. Importantly, the phosphorylation levels of S149, which is not phosphorylated by PfPKB, remained almost unaltered upon A443654 treatment (Fig. 4B). Based on these observations, PfPKB emerged as the likely parasite kinase needed for the phosphorylation of S103.

### Phosphorylation of PfGAP45 during parasite development

Having demonstrated the phosphorylation of PfGAP45 at S103 and S149, we next investigated if the phosphorylation of these sites is developmentally regulated. To this end, parasite lysates were prepared from late trophozoites (∼33 h post invasion) to late schizont/segmentor stage and were probed with phospho specific antibodies. While the phosphorylation of S149 was observed as early as ∼39 h, when the parasites were late trophozoites or early schizonts, phosphorylation of S103 was undetectable. In contrast, S103 phosphorylation was seen mainly at 42–45 h in mature schizonts (Fig. 5A, B). To correlate with S103 and S149 phosphorylation, the expression profiles of PfCDPK1 and PfPKB the two candidate kinases for these sites, were also determined. While PfCDPK1 was expressed early in parasite development, PfPKB was expressed mainly in the mature schizonts as also reported earlier (Fig. 5C) [26]. The expression levels of PfCDPK1 and PfPKB correlate well with the phosphorylation status of S103 and S149, respectively. These data strongly suggest that the phosphorylation of S103 and S149 and the expression of the two candidate kinases for these sites are differentially regulated during parasite development.

### PLC regulates the phosphorylation of both S103 and S149

The results described in Figure 1 indicate that PfGAP45 may be phosphorylated in response to PLC mediated calcium release. The phospho S103 and S149 antibodies were used to study if the phosphorylation at these sites was influenced by PLC-calcium signaling. The treatment with BAPTA-AM almost completely prevented the phosphorylation at S103 and S149, thereby providing strong evidence for the involvement of calcium dependent kinases in the phosphorylation of these residues. The PLC inhibitor, U73122, significantly reduced the phosphorylation of PfGAP45 at both S103 and S149 (Fig. 6, lane 3). Moreover, when a calcium ionophore ionomycin, which facilitates intracellular calcium release, was used in combination with U73122, a significant recovery in phosphorylation of both these sites was observed (Fig. 6, lane 5 v/s lane 3). These data suggested that PLC mediated calcium release may regulate kinases like PfPKB [21] and PfCDPK1 (Sharma and co-workers, unpublished results), which in turn may phosphorylate S103 and S149 of PfGAP45. In contrast, no significant change in the phosphorylation of S103 or S149 was caused by 8-Br-cADP-ribose or Dantrolene (Fig. 6, lane 7 and 8).

### Phosphorylation of S89, S103 and S149 may not be involved in PfGAP45 targeting and glideosome assembly formation

We investigated the role of phosphorylation in PfGAP45 cellular localization and association with the glideosome assembly. GAP45 has N-terminal myristoylation and palmitoylation signals which are necessary for its membrane targeting [13], [14]. mCherry or GFP fused to PfGAP45 C-terminal is targeted to the IMC [Gilberger and co-workers, manuscript under revision, [5]]. Therefore, parasite lines over expressing GAP45 with GFP fused to its C-terminus were generated. Immunofluorescence assays suggested that GAP45-GFP was targeted to the expected localization at the IMC as indicated by co-localization with endogenous PfGAP45 (Fig. S2, Fig. S3) as well as IMC protein PfMTIP (Fig. 7). The alanine or phosphate mimicking aspartic acid mutation of phosphorylation sites S89, S103 and S149 did not appear to cause significant changes to the co-localization with PfMTIP (Fig. 7, Fig. 3B) in late schizonts/merozoites. Cellular fractionation experiments were performed to further validate the membrane targeting of PfGAP45 and mutants in merozoites. The endogenous PfGAP45 was almost completely insoluble in PBS as well as high salt buffer. While it was readily soluble in Triton X-100, its solubility was extremely low in sodium carbonate pH 11.0 (Fig. 8). The mutation of any of the phosphorylation sites did not cause a discernable change in its solubility, therefore phosphorylation at the mutated sites may not play a direct role in membrane targeting of PfGAP45. In addition, ^35^S-labeling-immunoprecipitation and co-immunoprecipitation experiments suggested no significant change in the interaction of GAP45-GFP with other glideosome proteins upon mutation of S89, S103 and S149 (Fig. S4). Therefore, the phosphorylation of S89, S103 and S149 may not be involved directly in glideosome complex formation. It is indeed possible that the phosphorylation of PfGAP45 at additional sites is needed for its function.

## Discussion

It is clear that the assembly of glideosome complex proteins is pivotal for the targeting and functioning of the actomyosin motor in apicomplexans. Several studies performed on *Plasmodium* [5], [6], [8] and *Toxoplasma* [3] have indicated that the motor complex comprises of at least four major proteins MyoA, MLC1/MTIP, GAP45 and GAP50 with additional proteins associated with the complex [13], [33]. Although recent work suggests that GAP45 and MTIP may be phosphorylated [7], [16], [17], there is little information about what cellular events regulate their phosphorylation. Given the role of calcium and the importance of the motor in invasion, we dissected calcium signaling events that may control PfGAP45 phosphorylation. U73122 a pharmacological inhibitor of PLC which is also effective against malarial PLC [(Pushkar Sharma, Guru Sharma unpublished results), [34]], blocks intracellular calcium release in the parasite [35], [36], which has been implicated in RBC invasion [17]–[19]. In addition, cADP ribose dependent RyR calcium channels have also been implicated in invasion [20]. Depletion of free intracellular calcium using BAPTA-AM prevented PfGAP45 phosphorylation significantly, which was the first indication that PfGAP45 is phosphorylated in a calcium dependent manner in the parasite. In the same experiment, we demonstrated that U73122 blocked its phosphorylation suggesting that PLC mediated calcium release may be responsible for PfGAP45 phosphorylation. The concentration of U73122 used in these studies was 10 µM as RBC invasion is blocked at this concentration (Vaid and Sharma, unpublished results, [18], [19]. A previous study had suggested that PfGAP45 phosphorylation may not be dependent on calcium [20]. ProQ Diamond, a dye which is suggested to specifically stain phosphorylated proteins, was used in this work. We used traditional ^32^P labeling to detect the phosphorylated proteins. It is possible that the significantly higher sensitivity of ^32^P labeling over ProQ Diamond allowed the detection of differences in PfGAP45 phosphorylation in these studies. Moreover, we have used phosphorylation site specific antibodies to confirm PfGAP45 phosphorylation. Consistent with the earlier report [20], we did not find a significant difference in PfGAP45 phosphorylation when parasites were treated with Dantrolene and 8Br-cADP, which are the modulators of the RyR pathway in this process. The phosphorylation of PfGAP45 at S103 and S149 was almost completely abolished by BAPTA-AM (Fig. 6). Metabolic labeling (Fig. 1, Fig. S6) indicated that even though PfGAP45 phosphorylation was markedly reduced after BAPTA-AM treatment, the residual phosphorylation suggested that it may be phosphorylated at additional sites possibly in a calcium independent manner. We identified several PfCDPK1 sites on PfGAP45 *in vitro*. Interestingly, S103 was phosphorylated by both PfPKB and PfCDPK1 *in vitro*. To decipher, the likely candidate kinase for this site in the parasite, A443654, which was previously identified as a PfPKB inhibitor [17], was used. This compound blocked the phosphorylation of S103 and not S149, which is not a PfPKB site. Our observation that the phosphorylation of S103 is mediated via the PLC-calcium pathway fits in well with the previous finding which implicated PLC mediated calcium release in PfPKB activation [17]. Our preliminary studies have indicated that PLC may regulate PfCDPK1 in the parasite (Sharma, Thomas, unpublished results), which may possibly explain the PLC dependent phosphorylation of S149.

Our studies and those performed by Holder and co-workers (manuscript submitted), which involved a slightly different expression construct that had GFP between the globular domain and the coiled-coil domain, indicated that the phosphorylation at S89, S103 and S149 may not be essential for targeting of PfGAP45 to the IMC. However, the possibility of additional phosphorylation at sites, other than the ones mutated in this study [30], playing a role in this process can not be ruled out.

Despite subtle differences in the amino acid sequences of TgGAP45 and PfGAP45 (Fig. S5), it is interesting that TgGAP45 can be replaced by PfGAP45 [13]. While the function of PfGAP45 and TgGAP45 may be the same, it is possible that they may be regulated differently in the two parasites, which may be reflected by the differences in some of the phosphorylation sites in GAP45 from the two species. For instance, at least two of the three phosphorylation sites identified by us (S89 and S103) are not conserved in TgGAP45; the residue corresponding to S89 in PfGAP45 is E117 in TgGAP45, which may mimic a phosphoserine. It is possible that a negative charge may be necessary at this position in both TgGAP45 and PfGAP45, which in the case of PfGAP45 may be acquired as a result of phosphorylation. However, S103 is replaced by A131 in TgGAP45, which is a significant difference. Previously, S163 and S167 were reported to be phosphorylated in TgGAP45 and their dephosphorylation was considered important for interaction with TgGAP50 [15]. Although, this region of PfGAP45 and TgGAP45 is poorly conserved, there are several threonines in this region of PfGAP45, which may complement S163 and S167. It will be worth investigating if these threonines undergo phosphorylation-dephosphorylation in the case of PfGAP45. When this manuscript was in preparation, two proteomic studies on *P. falciparum* and *Toxoplasma gondii* proteins were published, which included the identification of phosphorylation sites on PfGAP45 [37] and TgGAP45 [16]. The studies on PfGAP45 supported our present work and suggest that S89, S103 and S149 are likely phosphorylation sites on PfGAP45. Of the sites identified on TgGAP45, only S169 and S184/185 was dependent on calcium [16]. S185 of TgGAP45 corresponds to S149 of PfGAP45, which we have identified as a calcium dependent phosphorylation site in PfGAP45. Interestingly, the residue corresponding to S184 in PfGAP45 is E148, which may mimic a phosphoserine. The phosphorylation sites identified on TgGAP45 in this study were mainly on the disordered region that separates coiled-coil and the C-terminal globular domain. Our results indicate that PfGAP45 is phosphorylated on the coiled-coil domain at S89 and S103. The mutation of these sites did not alter the localization or interaction of PfGAP45 with other glideosome proteins. It is possible that the presence of endogenous PfGAP45 does not allow the effect of any of the mutants to be dominant. Alternatively, the phosphorylation at these sites in combination with other sites may be needed for PfGAP45 function. Additional studies like the replacement of the endogenous PfGAP45 allele with these phosphorylation site mutants will be needed to address these issues.

## Supporting Information

Figure S1 **Generation of antisera that recognizes PfGAP45 phosphorylated at S103 and S149.** Antisera were generated in rabbits against phosphopeptides spanning S103 and S149 (for details see Materials and Methods). Recombinant PfCDPK1 was incubated with recombinant PfGAP45 or its S103A or S149A mutant in the presence or absence (negative control) of ATP in a kinase assay mix. Subsequently, the affinity purified antisera against phosphorylated version of S149 (a) or S103 (b) or against the recombinant PfGAP45 (c) was used for western blotting. Please note the mobility shift caused by the phosphorylation of PfGAP45 in panel c.
(PDF)

Figure S2 **PfGAP45 phosphorylation mutants co-localize with endogenous PfGAP45.** Immunofluorescence assays were performed using anti-GFP (green) and anti-PfGAP45 (red) antibodies to localize episomally expressing GAP45-GFP or its mutants and endogenous PfGAP45.
(PDF)

Figure S3 **Live Imaging of PfGAP45 phosphorylation site mutants.** **A.** Imaging of GFP in parasites over-expresssing PfGAP45-GFP or its variants was performed after labeling the nuclei with DAPI. **B.** Immunofluorescence assays were performed using anti-PfMTIP (green) and anti-PfGAP45 (red) antibodies on *P. falciparum* 3D7.
(PDF)

Figure S4 **PfGAP45 phosphorylation site mutants associate with the glideosome motor complex.** **A.** Phosphorylation at S89, S103 and S149 does not affect PfGAP45-GFP association with the glideosome complex. [^35^S] Met/Cys was used to metabolically label PfGAP45-GFP or mutant expressing lines at the schizont stage. Anti GFP antibody was used to immunoprecipitate GAP45-GFP or its mutants. IPs were electrophoresed and analyzed by autoradiography. The components of the motor complex that co-immunoprecipitated are indicated based on their molecular size [14], [17]. *Bottom panel*, Western blot was performed using anti-GFP antibody on the lysates used in the same IP-experiment to assess the expression levels GAP45-GFP. **B.** GAP45-GFP or its variants were immunoprecipitated from transgenic parasite lines and the IP was used for western blotting with anti-MTIP antibody. There was no significant difference in the amount of MTIP co-immunoprecipitated with wild type or mutant GAP45. *Bottom panel*, Western blot was performed using MTIP antibody on the lysates used in the IP-experiment from top panel.
(PDF)

Figure S5 **Clustal W alignment of PfGAP45 and TgGAP45 protein sequence.** Cyan arrows indicate PfGAP45 phosphorylation sites identified in the present study and blue arrows indicate the phosphorylation sites on TgGAP45 which were identified previously [15].
(PDF)

Figure S6 **PfGAP45 phosphorylation is regulated via the PLC pathway in the parasite.** [^32^P] orthophosphoric acid was used to metabolically label synchronized parasites treated with indicated compounds as described in Fig. 1A. Immunoprecipitation was carried out using anti-GAP45 antibody and the IPs were electrophoresed and analyzed by phosphorimaging.
(PDF)

## References

[1] World Health Organization. Global Malaria Program (2010). World malaria report 2010..

[2] Gaur D, Mayer DC, Miller LH (2004). Parasite ligand-host receptor interactions during invasion of erythrocytes by Plasmodium merozoites.. International journal for parasitology.

[3] Gaskins E, Gilk S, DeVore N, Mann T, Ward G (2004). Identification of the membrane receptor of a class XIV myosin in Toxoplasma gondii.. J Cell Biol.

[4] Keeley A, Soldati D (2004). The glideosome: a molecular machine powering motility and host-cell invasion by Apicomplexa.. Trends Cell Biol.

[5] Baum J, Richard D, Healer J, Rug M, Krnajski Z (2006). A conserved molecular motor drives cell invasion and gliding motility across malaria life cycle stages and other apicomplexan parasites.. J Biol Chem.

[6] Green JL, Martin SR, Fielden J, Ksagoni A, Grainger M (2006). The MTIP-myosin A complex in blood stage malaria parasites.. J Mol Biol.

[7] Green JL, Rees-Channer RR, Howell SA, Martin SR, Knuepfer E (2008). The motor complex of Plasmodium falciparum: phosphorylation by a calcium-dependent protein kinase.. J Biol Chem.

[8] Jones ML, Kitson EL, Rayner JC (2006). Plasmodium falciparum erythrocyte invasion: a conserved myosin associated complex.. Mol Biochem Parasitol.

[9] Yeoman JA, Hanssen E, Maier AG, Klonis N, Maco B (2011). Tracking Glideosome-associated protein 50 reveals the development and organization of the inner membrane complex of Plasmodium falciparum.. Eukaryot Cell.

[10] Daher W, Soldati-Favre D (2009). Mechanisms controlling glideosome function in apicomplexans.. Curr Opin Microbiol.

[11] Kato N, Sakata T, Breton G, Le Roch KG, Nagle A (2008). Gene expression signatures and small-molecule compounds link a protein kinase to Plasmodium falciparum motility.. Nat Chem Biol.

[12] Fauquenoy S, Hovasse A, Sloves PJ, Morelle W, Dilezitoko AT (2011). Unusual N-glycan structures required for trafficking Toxoplasma gondii GAP50 to the inner membrane complex regulate host cell entry through parasite motility.. Mol Cell Proteomics.

[13] Frenal K, Polonais V, Marq JB, Stratmann R, Limenitakis J (2010). Functional dissection of the apicomplexan glideosome molecular architecture.. Cell Host Microbe.

[14] Rees-Channer RR, Martin SR, Green JL, Bowyer PW, Grainger M (2006). Dual acylation of the 45 kDa gliding-associated protein (GAP45) in Plasmodium falciparum merozoites.. Mol Biochem Parasitol.

[15] Gilk SD, Gaskins E, Ward GE, Beckers CJ (2009). GAP45 phosphorylation controls assembly of the Toxoplasma myosin XIV complex.. Eukaryot Cell.

[16] Nebl T, Prieto JH, Kapp E, Smith BJ, Williams MJ (2011). Quantitative in vivo analyses reveal calcium-dependent phosphorylation sites and identifies a novel component of the Toxoplasma invasion motor complex.. PLoS Pathog.

[17] Vaid A, Thomas DC, Sharma P (2008). Role of Ca2+/calmodulin-PfPKB signaling pathway in erythrocyte invasion by Plasmodium falciparum.. J Biol Chem.

[18] Beraldo FH, Garcia CR (2005). Products of tryptophan catabolism induce Ca2+ release and modulate the cell cycle of Plasmodium falciparum malaria parasites.. J Pineal Res.

[19] Singh S, Alam MM, Pal-Bhowmick I, Brzostowski JA, Chitnis CE (2010). Distinct external signals trigger sequential release of apical organelles during erythrocyte invasion by malaria parasites.. PLoS Pathog.

[20] Jones ML, Cottingham C, Rayner JC (2009). Effects of calcium signaling on Plasmodium falciparum erythrocyte invasion and post-translational modification of gliding-associated protein 45 (PfGAP45).. Mol Biochem Parasitol.

[21] Vaid A, Sharma P (2006). PfPKB, a protein kinase B-like enzyme from Plasmodium falciparum: II. Identification of calcium/calmodulin as its upstream activator and dissection of a novel signaling pathway.. J Biol Chem.

[22] Trager W, Jensen JB (1976). Human malaria parasites in continuous culture.. Science.

[23] Struck NS, de Souza DS, Langer C, Marti M, Pearce JA (2005). Re-defining the Golgi complex in Plasmodium falciparum using the novel Golgi marker PfGRASP.. J Cell Sci.

[24] Wu Y, Sifri CD, Lei HH, Su XZ, Wellems TE (1995). Transfection of Plasmodium falciparum within human red blood cells.. Proc Natl Acad Sci U S A.

[25] Fidock DA, Wellems TE (1997). Transformation with human dihydrofolate reductase renders malaria parasites insensitive to WR99210 but does not affect the intrinsic activity of proguanil.. Proc Natl Acad Sci U S A.

[26] Kumar A, Vaid A, Syin C, Sharma P (2004). PfPKB, a novel protein kinase B-like enzyme from Plasmodium falciparum: I. Identification, characterization, and possible role in parasite development.. J Biol Chem.

[27] Boyle WJ, van der GP, Hunter T (1991). Phosphopeptide mapping and phosphoamino acid analysis by two-dimensional separation on thin-layer cellulose plates.. Methods Enzymol.

[28] Tonkin CJ, van Dooren GG, Spurck TP, Struck NS, Good RT (2004). Localization of organellar proteins in Plasmodium falciparum using a novel set of transfection vectors and a new immunofluorescence fixation method.. Mol Biochem Parasitol.

[29] Mamoun CB, Goldberg DE (2001). Plasmodium protein phosphatase 2C dephosphorylates translation elongation factor 1beta and inhibits its PKC-mediated nucleotide exchange activity in vitro.. Mol Microbiol.

[30] Winter D, Kugelstadt D, Seidler J, Kappes B, Lehmann WD (2009). Protein phosphorylation influences proteolytic cleavage and kinase substrate properties exemplified by analysis of in vitro phosphorylated Plasmodium falciparum glideosome-associated protein 45 by nano-ultra performance liquid chromatography-tandem mass spectrometry.. Anal Biochem.

[31] Marks AR (1997). Intracellular calcium-release channels: regulators of cell life and death.. Am J Physiol.

[32] Luo Y, Shoemaker AR, Liu X, Woods KW, Thomas SA (2005). Potent and selective inhibitors of Akt kinases slow the progress of tumors in vivo.. Mol Cancer Ther.

[33] Bullen HE, Tonkin CJ, O'Donnell RA, Tham WH, Papenfuss AT (2009). A novel family of Apicomplexan glideosome-associated proteins with an inner membrane-anchoring role.. J Biol Chem.

[34] Raabe AC, Wengelnik K, Billker O, Vial HJ (2011). Multiple roles for Plasmodium berghei phosphoinositide-specific phospholipase C in regulating gametocyte activation and differentiation.. Cell Microbiol.

[35] Gazarini ML, Thomas AP, Pozzan T, Garcia CR (2003). Calcium signaling in a low calcium environment: how the intracellular malaria parasite solves the problem.. J Cell Biol.

[36] Hotta CT, Markus RP, Garcia CR (2003). Melatonin and N-acetyl-serotonin cross the red blood cell membrane and evoke calcium mobilization in malarial parasites.. Braz J Med Biol Res.

[37] Treeck M, Sanders JL, Elias JE, Boothroyd JC (2011). The phosphoproteomes of Plasmodium falciparum and Toxoplasma gondii reveal unusual adaptations within and beyond the parasites' boundaries.. Cell Host Microbe.

