# Supplementary figures and images for: Regulation of Plasmodium falciparum Glideosome Associated Protein 45 (PfGAP45) Phosphorylation
Source: PLoS One. 2012 Apr 27;7(4):e35855. doi: 10.1371/journal.pone.0035855 (PMC3338798; doi:10.1371/journal.pone.0035855)

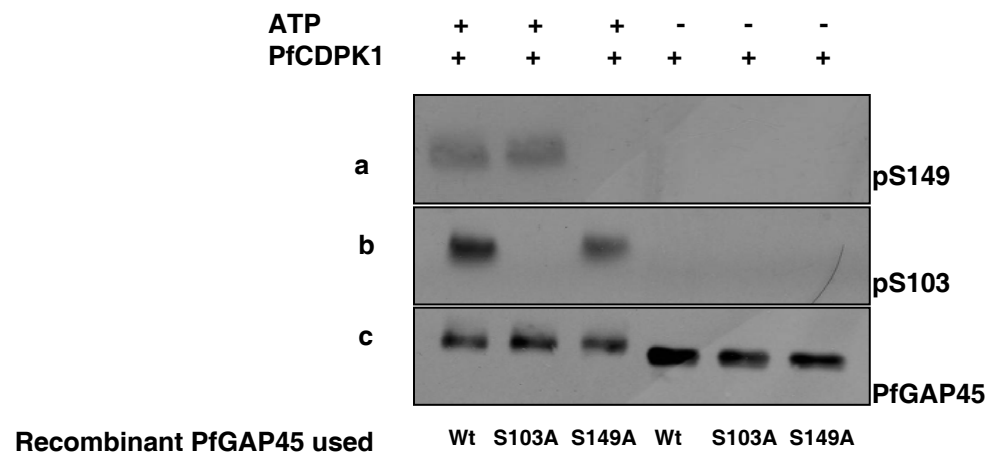

Phosphopeptides used for antisera

EPAHEE-**pS**-IYFTY (**S149**)

DLERS-**pS**-NDIYSES (**S103**)

Figure S1

Supplement: Figure S1 — Generation of antisera that recognizes PfGAP45 phosphorylated at S103 and S149. Antisera were generated in rabbits against phosphopeptides spanning S103 and S149 (for details see Materials and Methods). Recombinant PfCDPK1 was incubated with recombinant PfGAP45 or its S103A or S149A mutant in the presence or absence (negative control) of ATP in a kinase assay mix. Subsequently, the affinity purified antisera against phosphorylated version of S149 (a) or S103 (b) or against the recombinant PfGAP45 (c) was used for western blotting. Please note the mobility shift caused by the phosphorylation of PfGAP45 in panel c. (PDF) [file pone.0035855.s001.pdf]

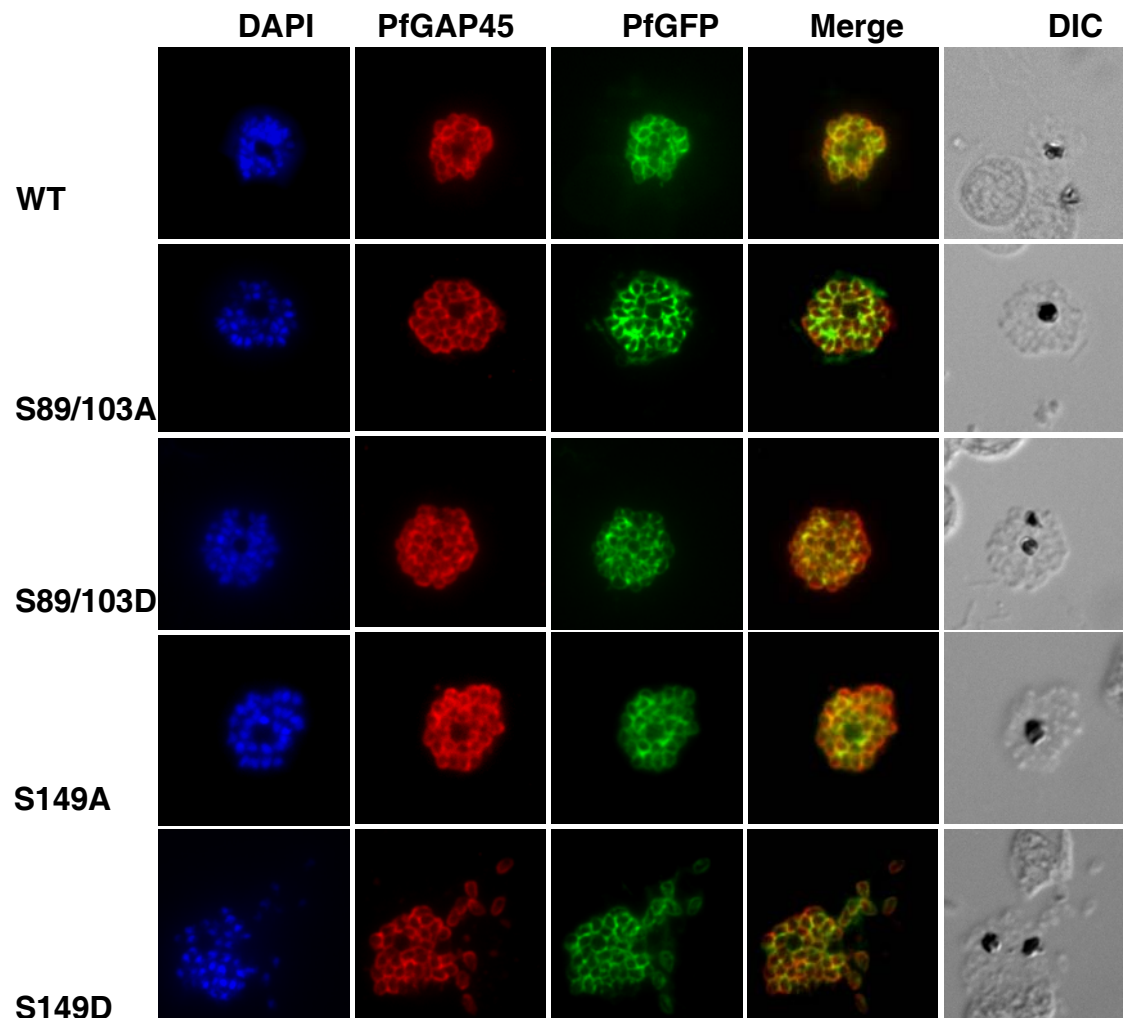

Figure S2

Supplement: Figure S2 — PfGAP45 phosphorylation mutants co-localize with endogenous PfGAP45. Immunofluorescence assays were performed using anti-GFP (green) and anti-PfGAP45 (red) antibodies to localize episomally expressing GAP45-GFP or its mutants and endogenous PfGAP45. (PDF) [file pone.0035855.s002.pdf]

**A****Wt**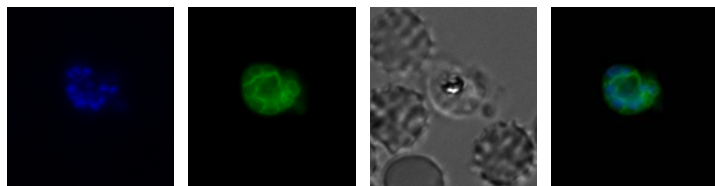**S89/103A**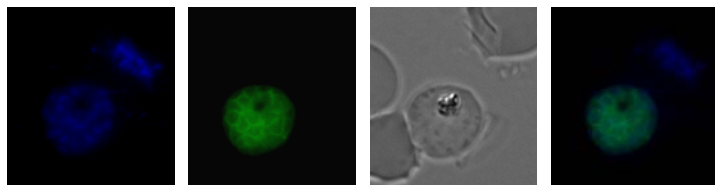**S89/103D**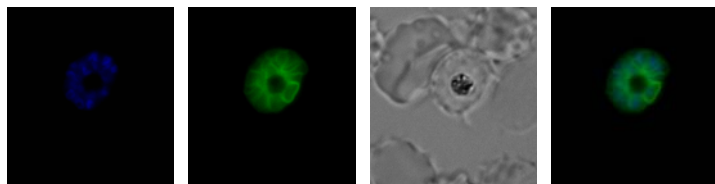**S149A**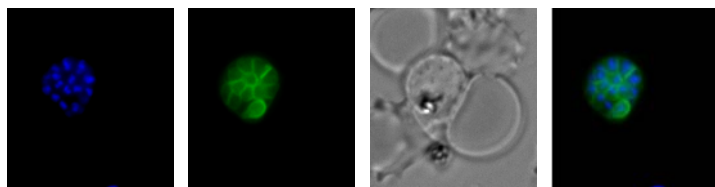**S149D**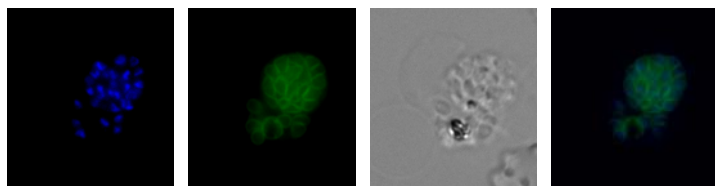**DAPI****GFP****DIC****Merge****B****DAPI****GAP45****MTIP****DIC****Merge**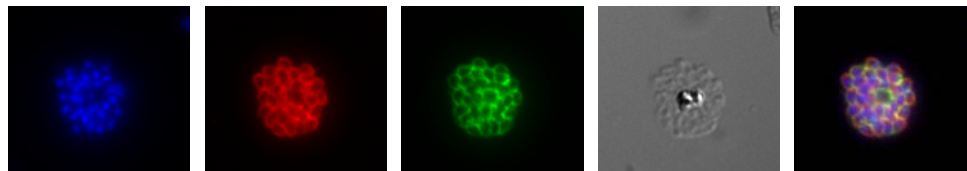**Figure S3**

Supplement: Figure S3 — Live Imaging of PfGAP45 phosphorylation site mutants. A. Imaging of GFP in parasites over-expresssing PfGAP45-GFP or its variants was performed after labeling the nuclei with DAPI. B. Immunofluorescence assays were performed using anti-PfMTIP (green) and anti-PfGAP45 (red) antibodies on P. falciparum 3D7. (PDF) [file pone.0035855.s003.pdf]

A

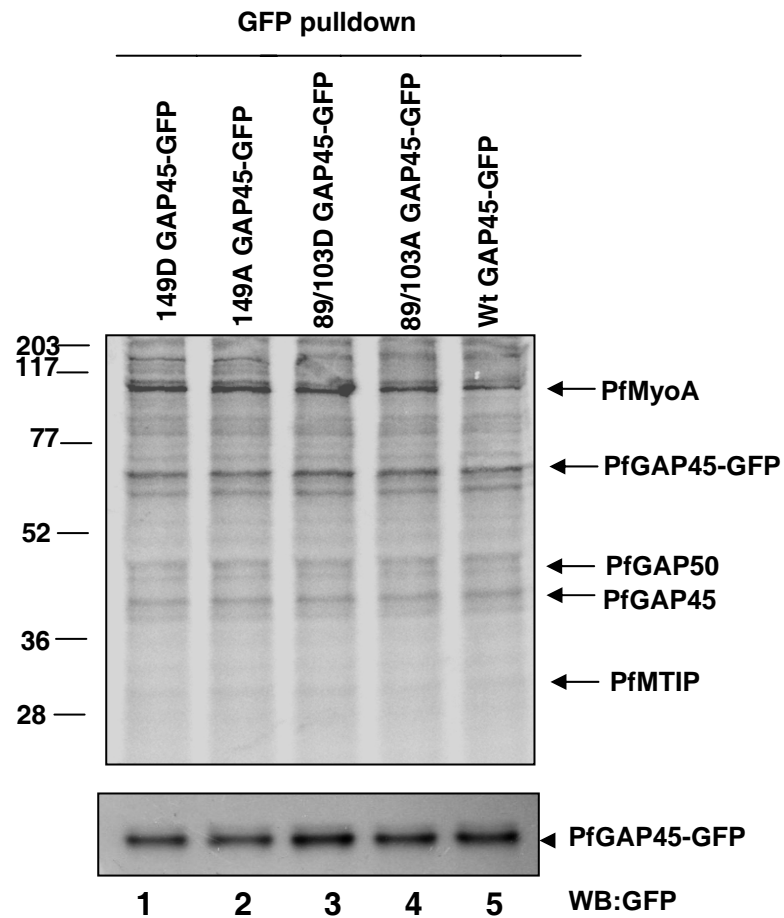

B

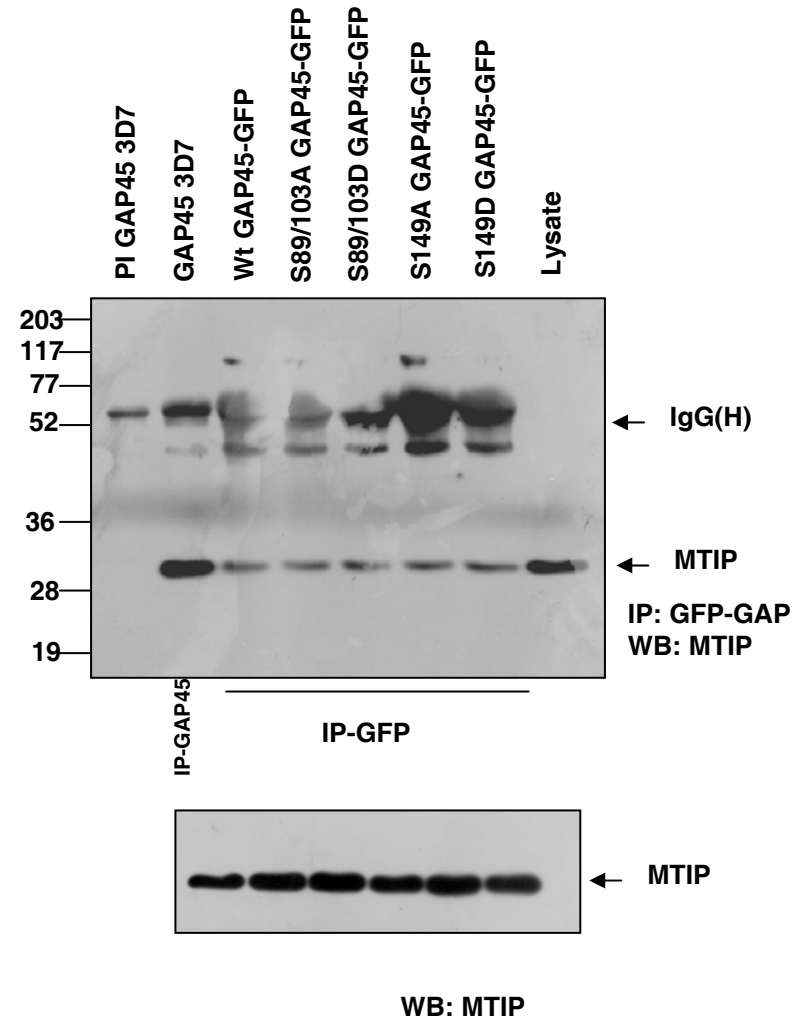

Figure S4

Supplement: Figure S4 — PfGAP45 phosphorylation site mutants associate with the glideosome motor complex. A. Phosphorylation at S89, S103 and S149 does not affect PfGAP45-GFP association with the glideosome complex. [35S] Met/Cys was used to metabolically label PfGAP45-GFP or mutant expressing lines at the schizont stage. Anti GFP antibody was used to immunoprecipitate GAP45-GFP or its mutants. IPs were electrophoresed and analyzed by autoradiography. The components of the motor complex that co-immunoprecipitated are indicated based on their molecular size [14], [17]. Bottom panel, Western blot was performed using anti-GFP antibody on the lysates used in the same IP-experiment to assess the expression levels GAP45-GFP. B. GAP45-GFP or its variants were immunoprecipitated from transgenic parasite lines and the IP was used for western blotting with anti-MTIP antibody. There was no significant difference in the amount of MTIP co-immunoprecipitated with wild type or mutant GAP45. Bottom panel, Western blot was performed using MTIP antibody on the lysates used in the IP-experiment from top panel. (PDF) [file pone.0035855.s004.pdf]

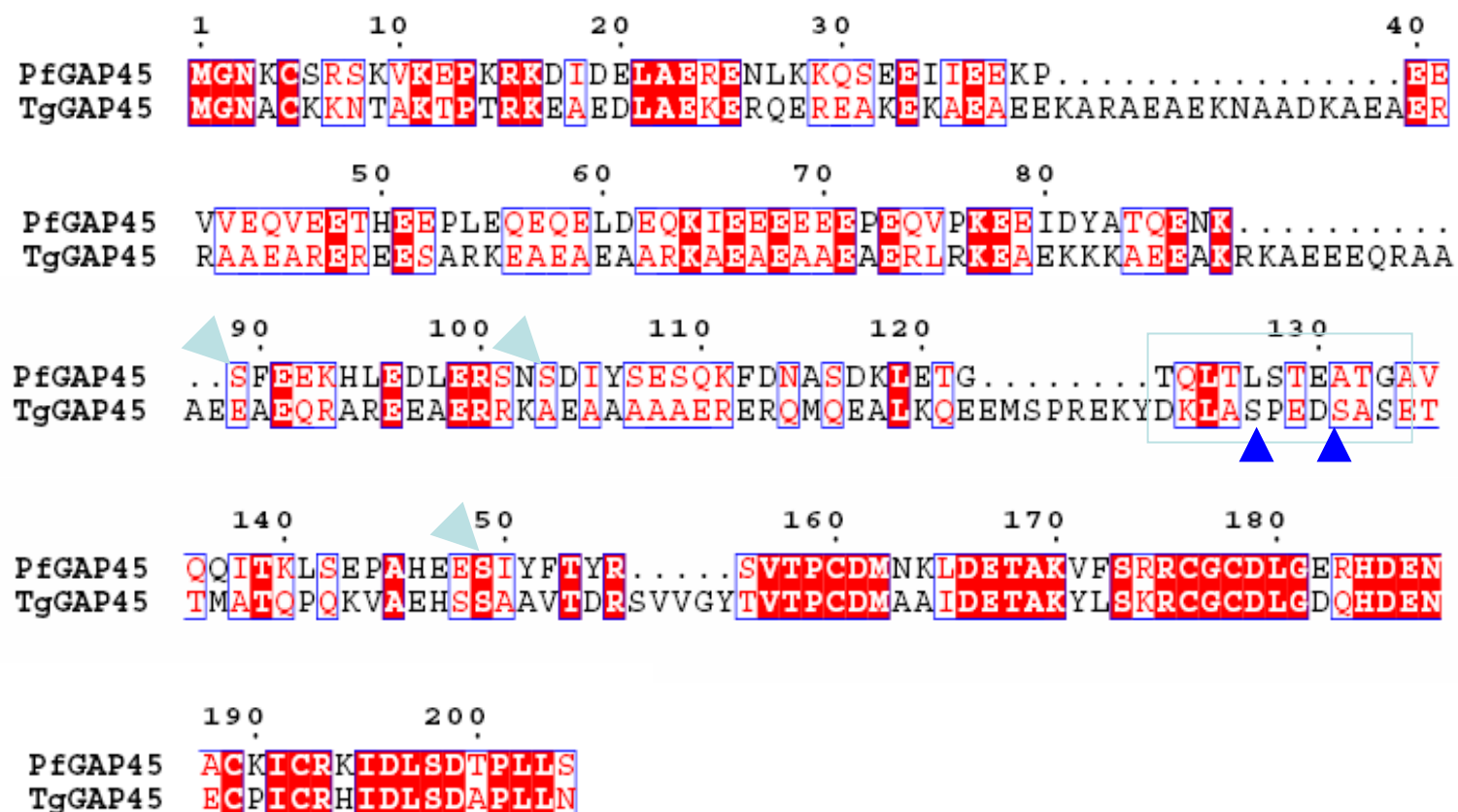

Figure S5

Supplement: Figure S5 — Clustal W alignment of PfGAP45 and TgGAP45 protein sequence. Cyan arrows indicate PfGAP45 phosphorylation sites identified in the present study and blue arrows indicate the phosphorylation sites on TgGAP45 which were identified previously [15]. (PDF) [file pone.0035855.s005.pdf]

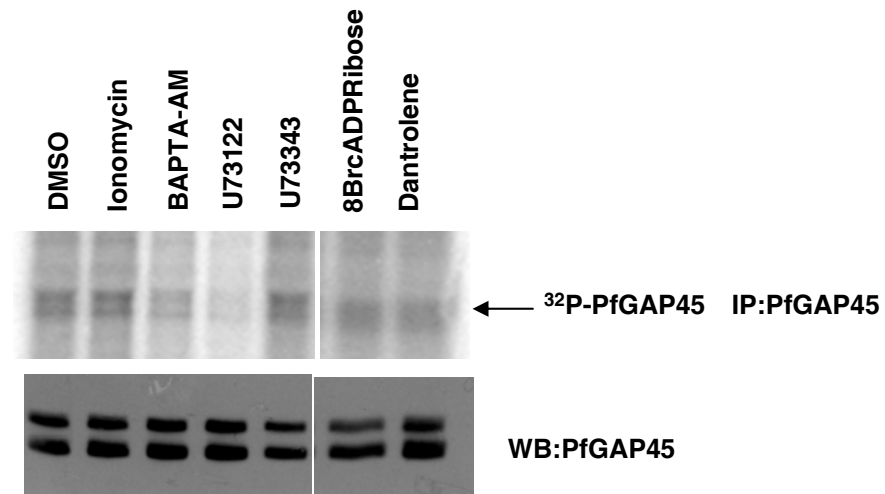

Figure S6

Supplement: Figure S6 — PfGAP45 phosphorylation is regulated via the PLC pathway in the parasite. [32P] orthophosphoric acid was used to metabolically label synchronized parasites treated with indicated compounds as described in Fig. 1A. Immunoprecipitation was carried out using anti-GAP45 antibody and the IPs were electrophoresed and analyzed by phosphorimaging. (PDF) [file pone.0035855.s006.pdf]
